# Dental peculiarities in the silvery mole-rat: an original model for studying the evolutionary and biological origins of continuous dental generation in mammals

**DOI:** 10.7717/peerj.1233

**Published:** 2015-09-10

**Authors:** Helder Gomes Rodrigues, Radim Šumbera

**Affiliations:** 1 Centre de Recherche sur la Paléobiodiversité et les Paléoenvironnements (CR2P), UMR CNRS 7207, Museum national d’Histoire naturelle, Université Paris 6, Paris, France; 2 Department of Zoology, Faculty of Science, University of South Bohemia, České Budějovice, Czech Republic

**Keywords:** Bathyergidae, Teeth, Morphology, Bone remodelling, Replacement, Drift, Resorption, Synapsids

## Abstract

Unravelling the evolutionary and developmental mechanisms that have impacted the mammalian dentition, since more than 200 Ma, is an intricate issue. Interestingly, a few mammal species, including the silvery mole-rat *Heliophobius argenteocinereus*, are able to replace their dentition by the addition of supernumerary molars at the back of jaw migrating then toward the front. The aim here was to demonstrate the potential interest of further studying this rodent in order to better understand the origins of continuous dental replacement in mammals, which could also provide interesting data concerning the evolution of limited dental generation occurring in first mammals. In the present study, we described the main stages of the dental eruptive sequence in the silvery mole-rat and the associated characteristics of horizontal replacement using X-ray microtomography. This was coupled to the investigation of other African mole-rats which have no dental replacement. This method permitted to establish evidence that the initial development of the dentition in *Heliophobius* is comparable to what it is observed in most of African mole-rats. This rodent first has premolars, but then identical additional molars, a mechanism convergent to manatees and the pygmy rock-wallaby. Evidence of continuous replacement and strong dental dynamics were also illustrated in *Heliophobius*, and stressed the need to deeply investigate these aspects for evolutionary, functional and developmental purposes. We also noticed that two groups of extinct non-mammalian synapsids convergently acquired this dental mechanism, but in a way differing from extant mammals. The discussion on the diverse evolutionary origins of horizontal dental replacement put emphasis on the necessity of focusing on biological parameters potentially involved in both continuous and limited developments of teeth in mammals. In that context, the silvery mole-rat could appear as the most appropriate candidate to do so.

## Introduction

Mammals differs from their extinct non-mammalian synapsid relatives by numerous anatomical, physiological and biological innovations (Kielan-Jaworowska, Cifelli & Luo, 2004; Kemp, 2005). Mammals notably acquired a more complex, but reduced dentition impacting both number of teeth and number of dental generations (Osborn & Crompton, 1971; Luo, Kielan-Jaworowska & Cifelli, 2004). Numerous studies attempted to study the biological and functional origins of the reduction of dental replacement in mammals (e.g., Luckett, 1993; Luo, Kielan-Jaworowska & Cifelli, 2004; Van Nievelt & Smith, 2005). In that context, the mouse, the traditional biological model, which has however no dental replacement, has appeared highly valuable to investigate the biological processes involved in dental development and replacement (e.g., Peterková, Lesot & Peterka, 2006; Jernvall & Thesleff, 2012; Peterková et al., 2014), also by means of genetically modified specimens developing supernumerary teeth (e.g., Järvinen et al., 2006; D’Souza & Klein, 2007; Juuri et al., 2013). Several developmental studies on other mammals, such as minipig, ferret and mainly humans, provided complementary data on tooth replacement (e.g., Järvinen, Tummers & Thesleff, 2009; Wang & Fan, 2011; Buchtová et al., 2012).

Continuous dental replacement (CDR) is very rare in mammals. It was first described in manatees (*Trichechus* sp.; Stannius, 1845) and in the pygmy rock-wallaby (*Petrogale concinna*; Thomas, 1904). This mechanism is described as a continuous development of teeth at the back of the jaw, then horizontally migrating toward the front, and replacing anterior cheek teeth shedding (Domning & Hayek, 1984). This kind of horizontal replacement differs from the continuous replacement of most vertebrates replacing their teeth locus by locus. The development of additional teeth distally reminds of a mechanism lost more than 200 Ma ago in first mammals (e.g., *Morganucodon*^†^; Luo, Kielan-Jaworowska & Cifelli, 2004). Consequently, investigating a mammalian model having a continuous replacement of its dentition would be optimal from evolutionary and developmental viewpoints. It would allow to compare the mechanisms involved in the continuous development of teeth in a few extant mammals, with those involved in the regression of the dental lamina, which prevents dental replacement and additional development of teeth distally in most mammals (Buchtová et al., 2012). By extension, it would also permit to better understand what it happened in first mammalian dentition more than 200 Ma ago.

Interestingly, CDR has also recently been described in rodents. It occurs in the silvery mole-rat, *Heliophobius argenteocinereus* (Gomes Rodrigues et al., 2011), which is an African rodent belonging to a strictly subterranean family, the African mole-rats (Bathyergidae). This species is solitary and occurs in a large area covering Central to Eastern Africa (Šumbera, Chitaukali & Burda, 2007). It lives in various environments including those with low food supplies and consisting of seasonally very hard soils, and also in rural areas where it frequently damages cultivated crops (Šumbera, Chitaukali & Burda, 2007). This species is not endangered, and could represent a suitable model for studying the origins of CDR. However, until now, breeding silvery mole-rat in captivity has failed outside of its area of distribution, probably because of lack of environmental stimuli, which remain to be discovered.

The system of CDR is slightly different in this mole-rat inasmuch as teeth are high-crowned, and the most anterior cheek teeth are totally resorbed inside the bone. Moreover, if the eruptive dental sequences of manatees and the wallaby are initiated by the development of premolars (Domning, 1982; Domning & Hayek, 1984; Sanson, 1980; Sanson, 1989), this process is still unknown in the silvery mole-rat, in which characteristics and dynamics of the dentitions remain to be accurately described. The silvery mole-rat and especially its relative, the naked mole-rat (*Heterocephalus*), represent the oldest bathyergid lineages (Patterson & Upham, 2014). The naked mole-rat lives in extended families and has a reduced tooth row with only three molars. As most mole-rats (including fossils) bear one premolar and three molars per tooth row, the most parsimonious hypothesis would suggest that extinct relatives of *Heliophobius* had the same dental formula. However, the functional origin of CDR in *Heliophobius* remains unclear, and we wanted to know if the setting up of this mechanism could be related to adaptive changes from a dentition originally reduced and therefore far less efficient than in other mole-rats, as supposed in *Heterocephalus* (Gomes Rodrigues, 2015). This reduced dentition assumed as a result of relaxed selection could have been associated with cooperative chisel-tooth digging (Gomes Rodrigues, 2015), whereas the CDR of *Heliophobius* could have been selected in relation to a putative higher solitary activity (i.e.; burrowing and foraging), especially in harder soils, inducing higher dental wear (Gomes Rodrigues et al., 2011).

More generally, the aim of this study is to more accurately describe the morphological characteristics of the dentition in the silvery mole-rat at different ontogenic stages, and to compared with other African mole-rats and mammals having CDR. These comparisons can provide important information on the initiation of the eruptive dental sequence of *Heliophobius*, on the putative maintaining of the system during the whole life of the animal, and on resulting effects on bone tissues. Because teeth are also migrating in the jaw, this implies bone remodelling in addition to resorption of dental tissues, as for other rodents showing mesial dental drift, but intensity is expected to vary in relation to the degree of migration (Gomes Rodrigues et al., 2012; Gomes Rodrigues, 2015). Then, we discussed the reliability of using this rodent as a future biological model for a better comprehension of the evolutionary origins of continuous dental generation in mammals and of associated underlying biological mechanisms.

## Material and Methods

**Figure 1:**
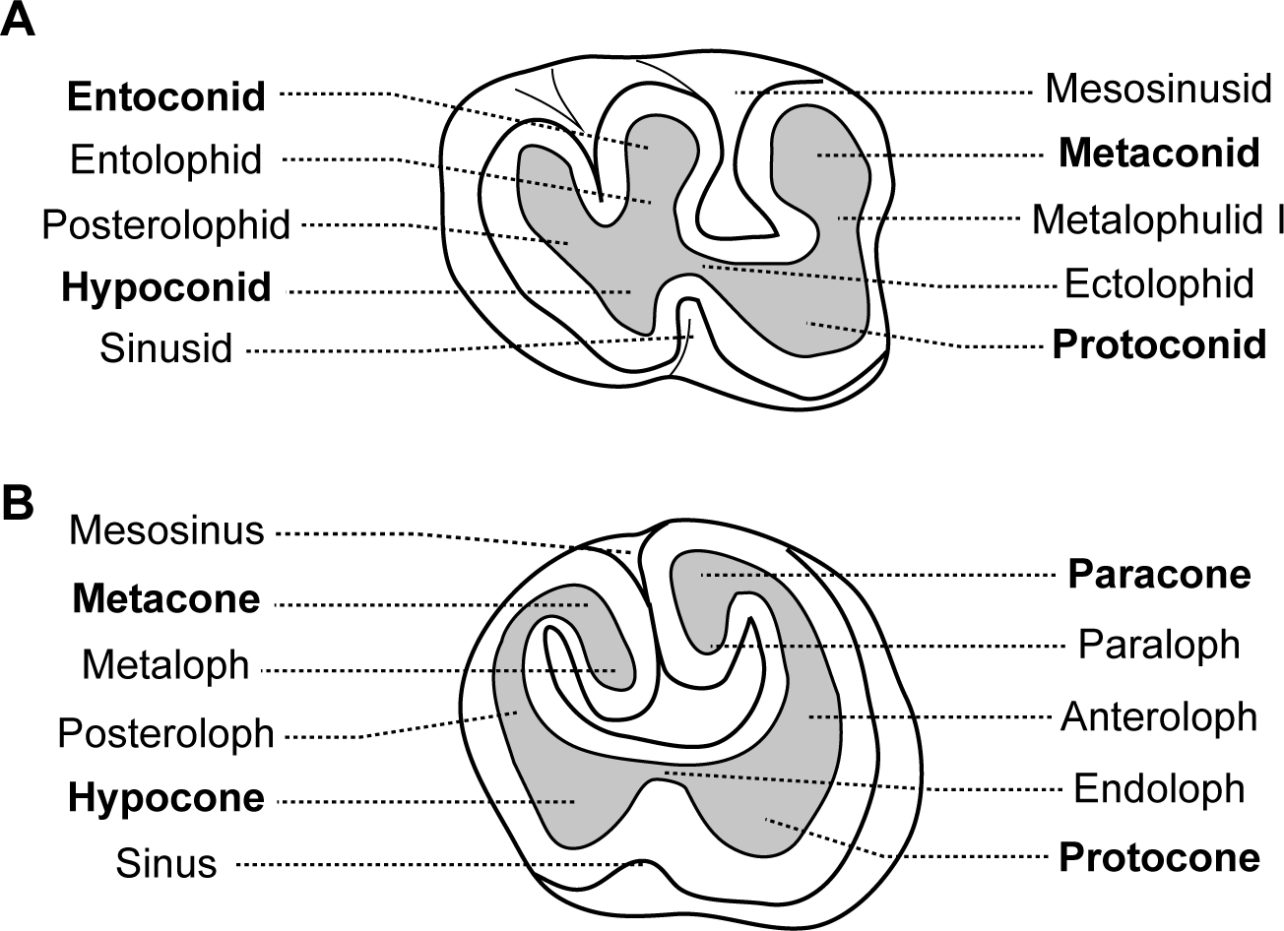
Dental nomenclature used for the Bathyergidae. Dental diagrams of right (A) lower and (B) upper molars of *Renefossor songhorensis* † (modified from Mein & Pickford, 2008).

Dental morphologies are difficult to describe in African-mole-rats because their teeth are rapidly worn and show a flat occlusal surface, which makes difficult putative homologies. As a result, dentition of very young specimens has been used when available to avoid such a problem and to draw reliable homologies, as initiated by Denys (1988) who described juveniles of African mole-rats (i.e.; a specimen of *Cryptomys* illustrated here, and a specimen of *Georychus*). Nine specimens of *Heliophobius argenteocinereus* were investigated using X-ray synchrotron and conventional microtomography (Table 1). Most individuals were born in captivity from wild females caught in 2000 and 2005, in Blantyre and Zomba area located in Southern Malawi. No animals were killed for the purpose of this study; we only used animals that died naturally. As a result, we did not have access to a complete ontogenetic series, and dental stages could not be chosen with high precision. Even if no experiment were conducted on living animals, all manipulations with captive animals were initially approved by the Control Commission for Ethical Treatment of Animals, University of South Bohemia, Faculty of Science, Czech Republic (17864/2005-30/300). Scanning of embryo and juvenile specimens (ID1bis, ID2bis, ID119, ID140, ID142) were realized at the European Synchrotron Radiation Facility (ESRF, Grenoble, beamline ID 19), with a pink beam at energy of 60 keV and using a cubic voxel of 7.46 µm. One adult specimen of *Heliophobius* (RMCA96.036-M-5467) was also scanned at the ESRF (see Gomes Rodrigues et al., 2011). Synchrotron microtomography has been proven to be very useful for precise imaging of small elements, such as teeth (Tafforeau et al., 2006). Other adult specimens (ID13, ID178, ID180) were scanned using a GE phoenix nanotom 180 at energy of 100 keV with a cubic voxel of 22.73 and 24.29 µm. Non-invasive virtual extractions of entire dentition (i.e., crown and roots) were realized with VG Studio Max 2.1 software (VG Studio Max, Heidelberg, Germany). Dentitions of juvenile specimens of other African mole-rats, such as *Cryptomys hottentotus* and *Georychus capensis* housed in the Museum National d’Histoire Naturelle of Paris (MNHN, France), and *Bathyergus janetta* housed in the National History Museum of London (NHM, United Kingdom), were analysed to draw comparisons (Table 1). Adult specimens of *Heterocephalus glaber* from the MNHN of Paris, and *Fukomys wandewostijneae*, *Georychus capensis* and *Bathyergus suillus* from the NHM of London were also considered (Table 1). Teeth were then described following the nomenclature of Wood & Wilson (1936) with a few modifications related to Bathyergidae (Fig. 1). *T^x^* refers to *x*th upper tooth, *T_x_* to *x*th lower tooth and *T_x_* to both *x*th upper and lower teeth. Alveolar dental walls of specimens of a pygmy rock wallaby from the Western Australian Museum of Perth (WAM, Australia), and of a manatee from the Poulard’s collection of the University of Lyon (France) were also investigated (Table 1).

**Table 1:** Absolute or relative age, and corresponding number of teeth per tooth row for investigated specimens. (x) means presence of not yet erupted tooth.

Family	Species	Specimen	Age	Number of cheek teeth
Bathyergidae	*Heliophobius argenteocinereus*	ID119	Embryo	(1)
		ID140	1 day	1(1)
		ID142	1 week	1(1)
		ID1bis	2 weeks	2
		ID2bis	2 months	2(1)
		ID13	2 years	4
		ID178	4 years	4(1)
		ID180	9 years	At least 4 and odontoma
		RMCA96.036-M-5467	Adult	5(1)
	*Heterocephalus glaber*	MNHN-ZM-MO 1884-1578	Adult	3
	*Bathyergus janetta*	NHM 47.7.8.26	Juvenile	2
	*Bathyergus suillus*	NHM 5.10.8.9	Adult	4
	*Georychus capensis*	MNHN-ZM-MO 1988-113	Juvenile	1(1)
		NHM 46.11.18.46	Adult	4
	*Cryptomys hottentotus*	MNHN-ZM-MO 1988-117	Juvenile	1(1)
	*Fukomys wandewostijneae*	NHM 39.291	Adult	4
Macropodidae	*Petrogale concinna*	WAM-M8499	Juvenile	4
Trichechidae	*Trichechus senegalensis*	M201	Adult	6(1)

## Results

### Ontogenic stages in the silvery mole-rat

**Figure 2:**
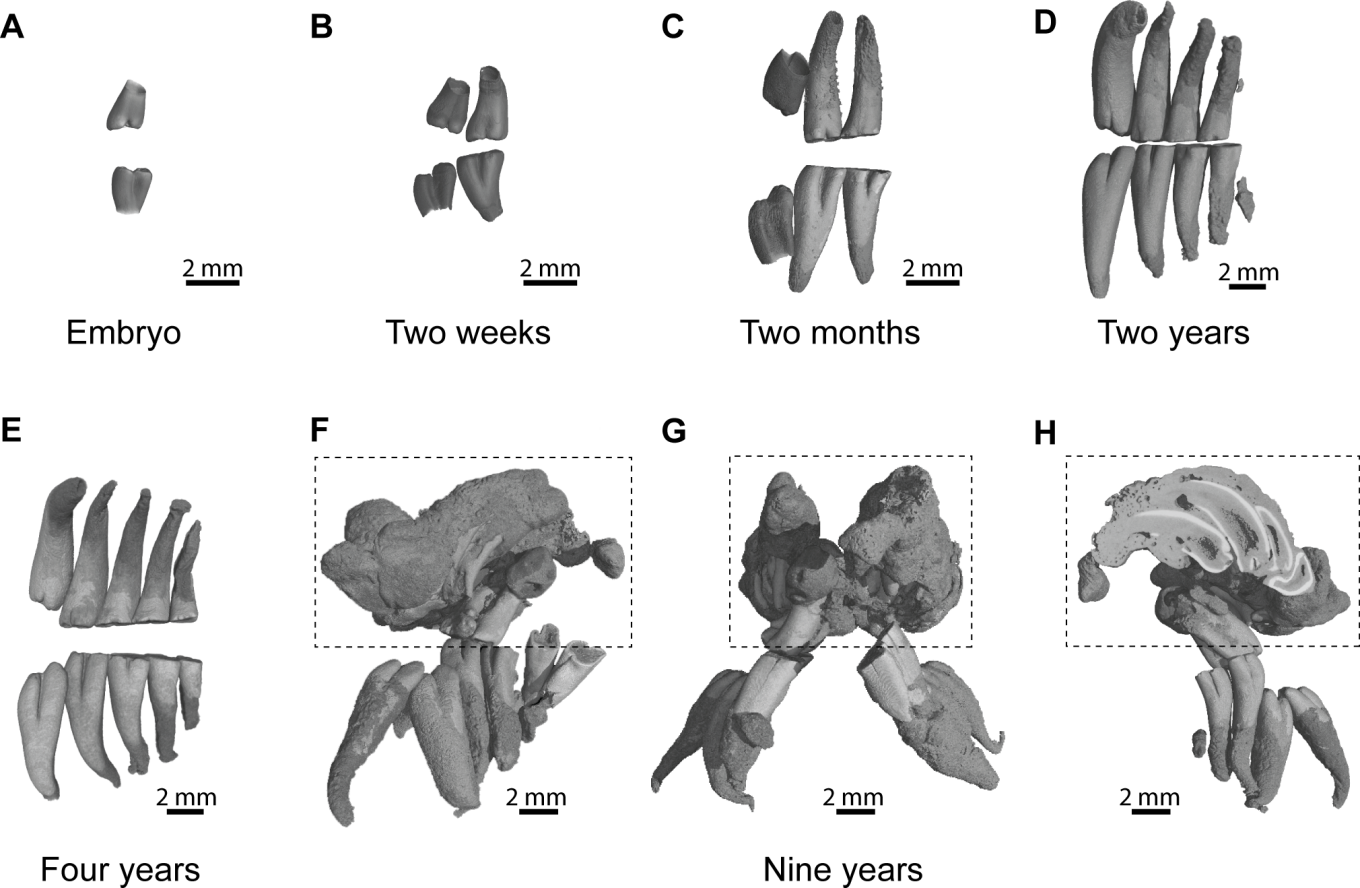
Dental ontogenic stages in the silvery mole-rat. (A–C) X-ray synchrotron microtomographic 3D rendering of dental rows of juveniles (ID119, ID1bis, ID2bis). (D–E) X-ray conventional microtomographic 3D rendering of dental rows of adult specimens (ID13, ID178). (F–H) X-ray conventional microtomographic 3D rendering of dental rows of the oldest specimen (ID180) showing a bilateral odontoma (dashed line square): (F) right lateral view, (G) anterior view, (F) lateral view showing a virtual section of the left odontoma.

An embryo of advanced stage of development shows one mineralized cheek tooth per dental row (Fig. 2A, ID119), while the second tooth is developing because a bony crypt can be observed. One day after birth (ID140), the second tooth starts to mineralized. One week later (ID142), the first tooth starts to erupt, and the second one is more developed but not yet covered by an enamel layer. A two-week-old specimen shows both teeth erupting with crown entirely mineralized, but with root still developing (Fig. 2B, ID1bis). After two months, the first teeth are worn and their root is entirely developed, and the third tooth is mineralizing and ready to erupt (Fig. 2C, ID2bis). The two-year-old and four-year-old specimens show the more frequently observed adult dentition, in having four functional teeth occluding with teeth still erupting distally (Figs. 2D–2E, ID13 and ID178). The first specimen shows remnants of teeth in resorption (Fig. 2D, ID13), while the mesial most teeth are highly reduced in the second specimen (Fig. 2E, ID178). The oldest specimen (nine-year-old) presents an abnormal dentition (Figs. 2F–2H, ID180). Upper and lower mesial-most teeth are normally developed, but present malocclusions and are ready to shed, although they are not strongly worn. Moreover, odontomas are formed in the distal part of the dentition and involved both upper dental rows. These odontomas are importantly developed, they spread in incisor alveoli, and include numerous malformed teeth, which subsequently appear in the virtual section (Fig. 2H).

### Dental morphology and eruptive sequence in the silvery mole-rat

#### Description

**Figure 3:**
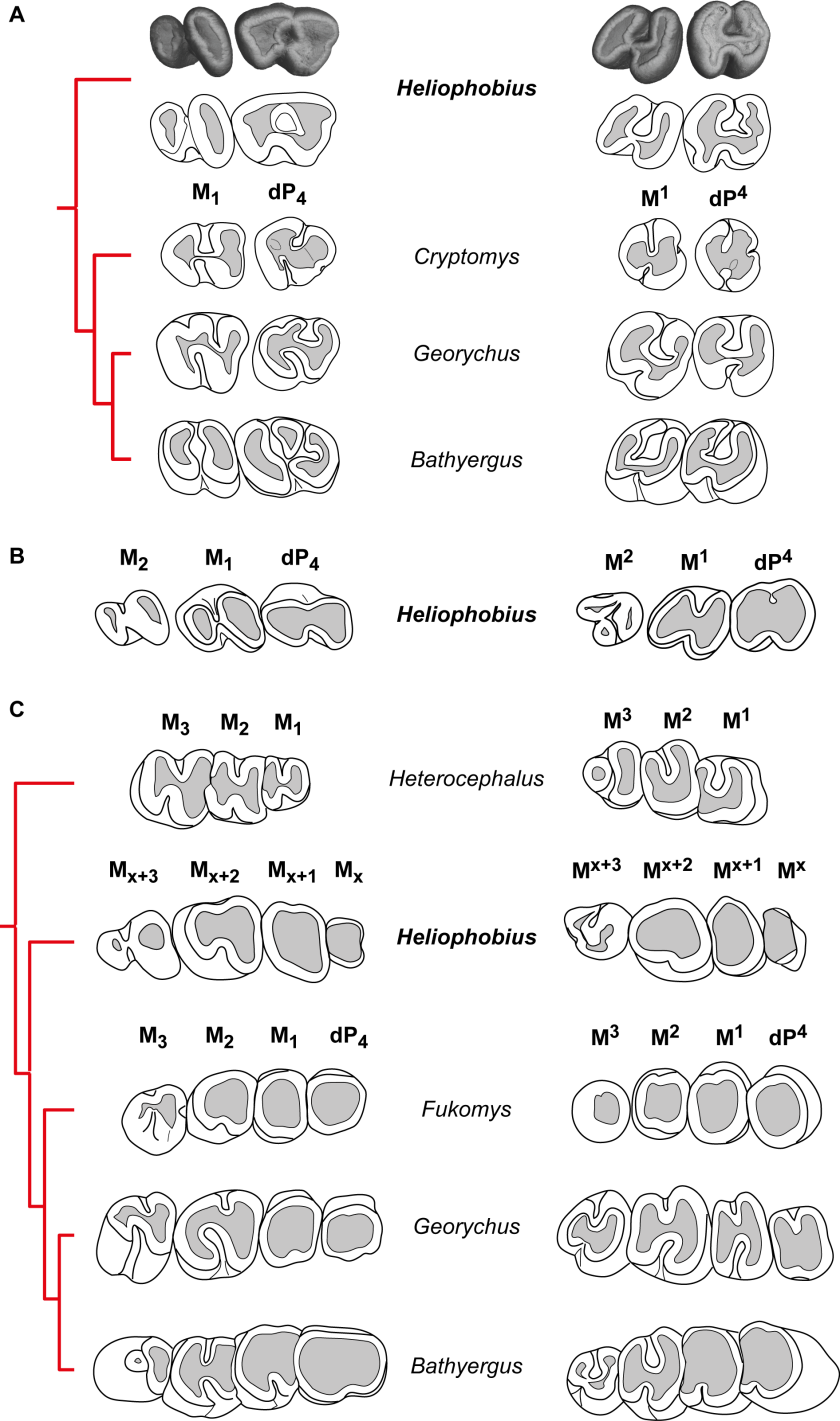
Dental eruptive sequence in the silvery mole-rat compared with other African mole-rats. (A) Comparisons of the morphology of first cheek teeth of juveniles represented by X-ray synchrotron microtomographic 3D rendering of a two-week-old silvery mole-rat (ID1bis) and by diagrams for African mole-rats (*Cryptomys hottentotus* MNHN-ZM-MO 1988-117, *Georychus capensis* MNHN-ZM-MO 1988-113, *Bathyergus janetta* NHM 47.7.8.26). (B) Diagrams of cheek teeth of a 2-month-old silvery mole-rat (ID2bis). (C) Diagrams of cheek teeth of a 2-year-old silvery mole-rat (ID13), and adult specimens of African mole-rats, such as *Heterocephalus glaber* (MNHN-ZM-MO 1884–1578), *Fukomys wandewostijneae* (NHM 39.291), *Georychus capensis* (NHM 46.11.18.46), *Bathyergus suillus* (NHM 5.10.8.9). Not to scale.

Cheek teeth of *Heliophobius* are very high-crowned, but their growth is limited since their single root is closed at the basal end (Fig. 2). As a result, both upper and lower teeth are cone-shaped. Unerupted teeth show enamel free areas (i.e., dentine can be already observed, Fig. 3A). Worn upper teeth shows heart-shaped occlusal surface, while lower teeth show pear-shaped occlusal surface (Fig. 3C).

- DP_4_ of *Heliophobius* is molariform (Fig. 3A). It shows a mesially located metaconid compared to the protoconid, which is less developed. The metalophulid I links these cusps. The metaconid also has a distal spur on the labial border, which reaches the entoconid and closes the mesosinusid. The ectolophid is marked in its mesial part linked to the protoconid, but it is interrupted to faintly marked at the level of the hypoconid. The sinusid is short and transverse. The posterolophid is strong and reaches the distal part of the entoconid. It can be assumed that the hypolophid transversally joins the hypoconid and the entoconid because of the crescent shape of the distal dentine area.
- M_1_ is clearly bilophodont, with an ectolophid missing to reduced, but more centrally located (Fig. 3A). As a result, the sinusid and the mesosinusid are equal in size. The mesial lophid consists of the protoconid and the metaconid connected by the metalophulid I. The distal lophid joins the reduced hypoconid to the entoconid. It is difficult to state if the hypolophid is present or fused with the posterolophid.
- M_2_ presents the same pattern, but the second lophid is much more reduced, and shows reduced entoconid and crest-like hypoconid (Fig. 3B). Following teeth present the same morphology as well, in having a highly reduced hypoconid (Fig. 3C).
- DP^4^ is molariform. The protocone is strong and linked to the paracone via the anteroloph. The paraloph is short and incipient and does not reach the protocone. Paracone and metacone are contiguous and close the mesosinus. The strong endoloph joins the protocone and the hypocone, which is mesio-distally compressed. The sinus is short and transverse. The hypocone is connected to the posteroloph, which distally joins the metacone. The metaloph is probably absent (Fig. 3A).
- M^1^ presents a very close pattern (Fig. 3A). However, the paraloph is absent, and the hypocone is not reduced.
- M^2^ shows a reduced protocone compared to the paracone (Fig. 3B). The anteroloph is weak to interrupted between these cusps. The distal part is much more reduced, with a small hypocone and a highly reduced and crest-like metacone. Following teeth have the same pattern (Fig. 3C).

#### Comparisons with other African mole-rats

- *Heliophobius* differs from all bathyergids in having additional molars. These teeth have highly reduced distal loph and lophids, and are like M3 of African mole-rats (Fig. 3C). However, it seems inappropriate to draw reliable comparisons between teeth whose homology is not established even if they are located at the same loci. *Heliophobius* also present the highest crown in association with *Bathyergus*.
- *Heliophobius* differs from *Heterocephalus* in having DP4, reduced distal lophid on M_1_ and M_2_ with a weaker ectolophid, more elongated lower molars, and in showing adjacent paracone and metacone closing the mesosinus in upper molars. Consequently, *Heliophobius* presents worn upper teeth with heart-shaped occlusal surface, whereas *Heterocephalus* presents worn upper teeth with U-shaped occlusal surface. Some specimens of *Heterocephalus* also have highly reduced to missing M3.
- *Heliophobius* differs from *Cryptomys* and *Fukomys* in having more elongated and less rounded cheek teeth, but is similar in having a simplified dental pattern with rapidly poorly distinct loph and lophids due to wear. *Heliophobius* differs from *Cryptomys* in having a metaconid less protruding mesially, a sinusid less protruding distally, and a hypoconid not reduced on DP_4_, and in having an ectolophid weaker on lower teeth. It also has a rounded mesial border without disruption between the paracone and the metacone on upper teeth, associated with a wider mesosinus because of a shorter paraloph (probably connected to the endoloph in *Cryptomys*).
- One of the main differences between *Heliophobius* and *Georychus* is about lower teeth. If the ectolophid is weak in *Heliophobius*, it is strong and distally connected to the entoconid in *Georychus*, and consequently the sinusid is larger and protruding distally. On DP_4_, the mesosinusid is closed in *Heliophobius*, whereas it is open in *Georychus*. On upper teeth, *Heliophobius* differs from *Georychus* in having a transverse sinus which is not mesially protruding, in having contiguous paracone and metacone on DP^4^, and a more reduced metacone on M^2^. *Heliophobius* presents worn upper teeth with heart-shaped occlusal surface, while *Georychus* presents worn upper teeth with an H-shaped occlusal surface.
- *Heliophobius* differs from *Bathyergus* in having a metaconid bigger than the protoconid and slightly protruding mesially, in having uninterrupted ectolophid and hypolophid, and a posterolophid connected to the entoconid on DP_4_. If the ectolophid is also weaker to discontinuous in molars of both genera, this lophid is more centrally located on M_1_ of *Heliophobius*, in which the hypoconid is more reduced and sinusid and mesosinusid are more open. Upper teeth of *Heliophobius* and *Bathyergus* are morphologically very close. However, the hypocone is more reduced on the DP^4^ of *Heliophobius*, the paraloph is slightly distinct, and paracone and metacone are closer.

### Characteristics of dental resorption and bone remodelling

**Figure 4:**
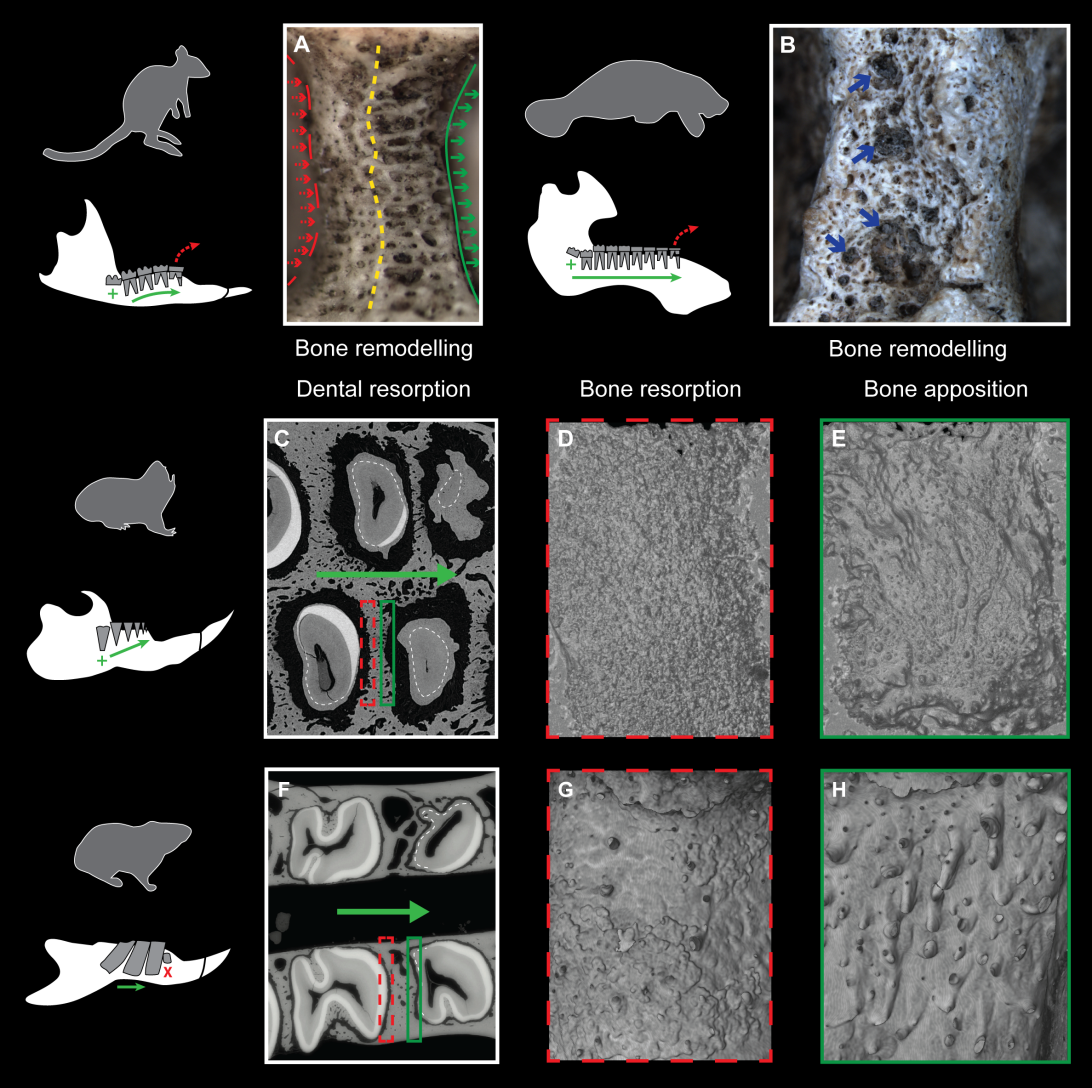
Consequences of dental mesial drift on bone tissues in the pygmy rock-wallaby, the manatee, and the silvery mole-rat compared with the felou gundi. (A) Picture of bone remodelling in the mandibular alveolar dental wall of a pygmy rock-wallaby (WAM-M8499). Green arrows associated with the continuous line indicate bone apposition and dashed red arrows associated with the dashed line indicate bone resorption. See the more compacted bone tissue on the left side and numerous openings of vascular channels on the left side of the alveolar wall. (B) Picture of bone remodelling in the mandibular alveolar dental wall of a manatee (M201). Blue arrows indicate a few openings of vascular channels. X-ray synchrotron microtomographic study of a specimen of (C–E) a silvery mole-rat (RMCA96.036-M-5467) and of (F–H) a felou gundi (MNHN-ZM-MO 1989-22; modified from Gomes Rodrigues et al., 2012). (C, F) Virtual cross-section of upper dental rows. The white dash line indicates the limit between the cementum layer and the teeth. The large green arrows indicate the direction of dental drift, and their length shows the degree of dental migration. (D, G) 3D rendering of the distal part of the alveolar wall showing an etched surface corresponding to bone resorption (dashed red boxes). (E, H) 3D rendering of the mesial part of the alveolar wall showing openings of vascular channels corresponding to bone apposition (green boxes). Not to scale.

Evidence of continuous mesial drift can be observed in bone tissues for specimens of a pygmy-rock wallaby, a manatee and a silvery mole-rat (Figs. 4A–4E). In the wallaby, the interalveolar septum presents a distally compacted aspect resulting from bone resorption, while the mesial part is illustrated by a high number of openings of vascular channels due to bone apposition (or formation, Fig. 4A), clearly observed in the manatee (Fig. 4B). In virtual cross-section of upper cheek teeth in the silvery mole-rat, bone resorption is shown on mesial side of teeth by the serrated aspect of the distal part of interalveolar septa, and by the corresponding alveolar wall showing a surface strongly etched (Figs. 4C–4D). Bone apposition is conversely illustrated on distal side of teeth by the presence of numerous openings of vascular channels in bone septa and alveolar surface (Figs. 4C and 4E). In this area, dental resorption also occurs because the enamel layer is reduced to absent and the outline is irregular due to the indirect compressive force of erupting distal molars also leading to periodontium disruption. Dental resorption is strongly efficient at the mesial-most side of the cheek tooth row, where the layer of cementum tends to be thicker (Fig. 4C).

## Discussion

### Normal initial dental development

**Figure 5:**
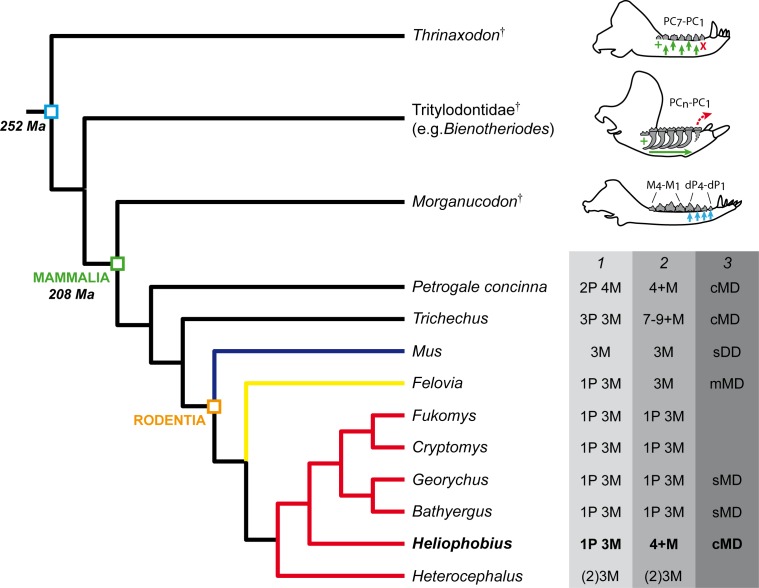
Phylogeny showing the dental characteristics and dynamics of some synapsids. Simplified diagrams of mandible showing the different types of cheek tooth replacement: continuous and alternate vertical replacement (small green arrows in *Thrinaxodon*^†^), continuous horizontal replacement (large green arrow in Tritylodontidae^†^), single and sequential vertical replacement (blue arrows in *Morganucodon*^†^). PC, post-canines; dP, deciduous premolars; M, molars. 1. Dental formulae in juveniles. 2. Dental formulae in adults. P, maximal number of premolars per tooth row; M, maximal number of molars per tooth row. 3. Mode of dental drift. sDD, slight distal drift; sMD, slight mesial drift; mMD, moderate mesial drift; cMD, continuous mesial drift. Blue branch, Muridae; yellow branch, Ctenodactylidae; red branch, Bathyergidae.

Before developing supernumerary molars, juveniles of manatees and of the pygmy rock-wallaby show a dental eruptive sequence similar to other mammals in having deciduous premolars before molars (Sanson, 1980; Domning, 1982; Gomes Rodrigues et al., 2011; Fig. 5). One of the major constraints when studying the dentition of the silvery mole-rat is the very high wear, which does not permit accurate morphological descriptions. The investigation of very young specimens helps us to determinate the eruptive sequence of this rodent. As a result, the crown morphology of both first lower and upper erupted cheek teeth strongly looks like DP4 of other African mole-rats. In DP^4^ of *Heliophobius*, the metaconid is more mesially located as in *Cryptomys* and *Georychus*, and the pattern is more complex than molars, which have a marked bilophodont structure, as in *Bathyergus*. The difference between DP_4_ and lower molars is more subtle, but the paraloph is present as in *Cryptomys*, and the second loph (hypocone-posteroloph-metacone) is not reduced. This means that the initial development of the cheek tooth row is comparable to what it is observed in most Bathyergidae, and in mammals with CDR as well (Fig. 5). These results also corroborate the hypothesis of Landry (1957), who proposed that the fourth premolar is coupled with molars and “that the extra teeth in *Heliophobius* represent entirely new dental elements added on to the end of the usual mammalian series” (p. 68).

*Heliophobius* has an eruptive dental sequence which differs from *Heterocephalus*, due to the occurrence of premolars, as seen in first known bathyergids from the Miocene of Africa (Mein & Pickford, 2008). *Heterocephalus* and *Heliophobius* clearly  display derived but opposite dental evolutionary trends, involving dental reduction in the oldest bathyergid lineage (only three or two molars, Fig. 5), and development of additional teeth in the lineage of *Heliophobius*. Data on extinct relatives of *Heliophobius* are thus essential to assess the evolutionary origins and the potential adaptive nature of this mechanism. More precisely, knowledge of the climatic, environmental and geological contexts relative to the appearance of CDR is needed. In the meantime, the functional characteristics of CDR in *Heliophobius* also remain to be precisely described *in vivo*, inasmuch as its dentition including originally four high-crown cheek teeth seems already highly efficient compared with other mole-rats, having higher bite force at molars, while digging with their incisors (e.g., *Fukomys*; Van Daele, Herrel & Adriaens, 2009).

The timing of dental eruptive sequence of *Heliophobius* is also very close to the timing known in *Georychus*, in which a new born has one teeth, a two-week-older specimen has two erupted teeth, and a four-week specimen has three erupted teeth (Taylor, Jarvis & Crowe, 1985). Unfortunately, we have no data concerning the development of the fourth teeth in *Heliophobius*, which might be tightly associated with the development of additional molars. It can be only observed that M2 and subsequent molars display a highly reduced distal parts. The additional molars are very similar to the penultimate molar, as observed in wallabies, and as noticed by Domning (1982) in manatees: “Trichechids were turning out unlimited numbers of “second molars” …” (p. 609). More material, as well as further developmental analyses are needed to precisely describe the formation of supernumerary teeth in *Heliophobius*. More generally, presence of premolars rapidly replaced by additional molars is another evidence of close convergent dental evolution between very different mammals, involving a rodent, a sirenian, and a macropod which had to face abrasive diet and/or terrigenous matter (Domning, 1982; Sanson, Nelson & Fell, 1985; Gomes Rodrigues et al., 2011; Šklíba, Šumbera & Vítámvás, 2011), probably just after weaning.

### Evidence of continuous dental replacement

One of the most interesting point concerns evidence of CDR in *Heliophobius*. Landry (1957) proposed that extra teeth can be present in this rodent, while Gomes Rodrigues et al. (2011) went further and proposed a CDR by analogy with what happens in manatees and the pygmy rock-wallaby. But, here, the study of the oldest specimen provides another evidence of continuous generation of teeth. The presence of bilateral odontomas in the distal part of the dentition in a rodent specimen supposed to have its last molars erupted for a while (e.g., between one and two years in *Georychus*; Taylor, Jarvis & Crowe, 1985) is intriguing, if we are not aware of the possibility of continuous dental development in supposed diphyodont animals. But, in the case of mammals with CDR, it can be related to developmental disruption probably linked to ageing. This was observed in a few specimens of old mice having bilateral odontomas at the level of the proximal end of incisors, in relation to their continuous growth (Dayan et al., 1994). This is obviously an evidence of the maintenance of active dental stem cells at the back of jaw. As incisors were also involved in this disorder and because their developmental origin is back and very close to last erupting molars (i.e., in outgrowth of pterygoid processes), a possible molecular interaction between incisor and molar tissues, which regulates their continuous activity, cannot be rejected and remains to be investigated. In any event, such results validate the hypothesis of continuous dental generation in the silvery mole-rat. Only when its breeding will be possible, precise and detailed investigation of mechanisms of dental development would permit to test the assumption of an extension of the dental lamina allowing CDR, conversely to its regression in diphyodont mammals, in which part of developmental mechanisms have already been described at the histological level (Buchtová et al., 2012).

### Evidence of intense dental drift

Three stages of dental mesial drift (or physiological drift) were defined in rodents (Fig. 5). The first one, observed in *Georychus* and *Bathyergus*, corresponds to a slight dental displacement and does not involve any dental loss (Gomes Rodrigues et al., 2011; Gomes Rodrigues, 2015; Fig. 5). The moderate mesial drift is present in all gundis (Ctenodactylidae), which only lose anterior teeth under the pressure of erupting posterior teeth (Gomes Rodrigues et al., 2012; Figs. 4 and 5). The strong or continuous mesial drift is only known in the silvery mole-rat because of the CDR leading to the constant loss of teeth at the front of the jaw. This dental dynamic involves bone remodelling and resorption of dental tissues whose intensity increases according to the degree of dental drift. This is evidenced when comparing alveolar bone characteristics between gundis (e.g., *Felovia*, Figs. 4F–4H; Gomes Rodrigues et al., 2012) and the silvery mole-rat. This latter shows a higher number of openings of vascular channel when bone is forming, and a more intensively etched surface when bone is in resorption, as similarly does the pygmy rock wallaby and the manatee. Moreover, alveolar septa are thinner in *Heliophobius* compared to other mole-rats in relation to constant bone remodelling (Gomes Rodrigues, 2015). Mesial teeth are highly resorbed and constantly covered by a thick layer of cementum, which might correspond to cementum repair protecting teeth (Jäger et al., 2008), necessary to maintain anchoring of the teeth in the dental alveolus via the periodontal ligament (Saffar, Lasfargues & Cherruau, 1997; Fleischmannová et al., 2010). Cemental modifications have also been reported in manatees (Miller, Sanson & Odell, 1980). The constant formation of teeth at the back of the jaw drives intense compressive forces which induce strong physical constraints on the whole dentition and on surrounding tissues (periodontium, alveolar bone). Nonetheless, dental disorders and misalignments are rare, because of the presence of a diastema in front of cheek teeth in these mammals.

The development and functionalization of the tooth in time and space occurred in close relation with the development of the surrounding bone and periodontal tissues. Moreover, the homeostasis of the alveolar bone is characterized by a high turn-over during the life of a tooth and by specific interactions with the periodontal ligament (Fleischmannová et al., 2010), which are modified during dental drift. The impact of dental drift has only been studied in the context of orthodontic researches in rat and mouse (e.g., Ren, Maltha & Kuijpers-Jagtman, 2004; Yoshimatsu et al., 2006), in which the migration is activated by an experimental system producing tensile forces (Wise & King, 2008). However, rat and mouse were rather described as having a slight distal drift (Johnson & Martinez, 1998; Fig. 5), without any detail on the cause of migration. The silvery mole-rat is pertinent for investigating the effect of the permanent migration of teeth on surrounding tissues involving notably homeostasis of alveolar bone and the periodontium, and also cementum repairs. The accurate description of the dental dynamics and its impact on both teeth and bone at the histological level in the silvery mole-rat and in rodents showing different degrees of drift will permit to comparatively assess the effects of natural migration and the associated mechanical constraints sustained by the whole masticatory apparatus. This could provide new insights on the functional properties of these various masticatory apparatus in relation to the ecology of these rodents.

### Evolutionary insights on the horizontal CDR

Interestingly, if the silvery mole-rat, manatees, and the pygmy rock-wallaby, represent unique cases of CDR in mammals, such an extended horizontal replacement has also been reported in non-mammalian synapsids (i.e., Traversodontidae^†^: Hopson, 1971; Liu, Bento Soares & Reichel, 2008; Liu & Sues, 2010; Tritylodontidae^†^: Kühne, 1956; Cui & Sun, 1987). These Mesozoic forms were assumed as herbivores and ingested fibrous plants (Kemp, 2005), probably also abrasive. This is more obvious in multi-cuspate tritylodontids (Fig. 5), especially derived taxa, which have higher dental wear in the mesial part of the dentition, and show evidence of dental drift and also dental resorption similar to what happen in manatees (Kühne, 1956; Cui & Sun, 1987; Jasinovski & Chinsamy, 2012).

The central point is that these synapsids acquired an extended or continuous horizontal replacement of more complex and larger teeth combined with the progressive loss of anterior post-canines. In addition, this sequential replacement was superimposed to the initial alternate and vertical replacement known in most non-mammalian synapsids (e.g., *Thrinaxodon*^†^, Fig. 5), which were not limited to two dental generations contrary to mammals (Hopson, 1971; Osborn & Crompton, 1971). Hopson (1971) partly explained the reduction of the number of dental generation in mammals, by the presence of more complex teeth anchored in a socket via periodontal ligaments (i.e., thecodonty) allowing to cope with mechanical stresses resulting from strong dental pressure during occlusion, which in contrast makes them more difficult to replace. In that context, eruption of teeth at the back, drifting then along the jaw, appears as a more suitable means to renew the dentition, inasmuch that such displacement can be favoured by transseptal fibers linking adjacent teeth, as noticed by Miller, Sanson & Odell (1980) in manatees, and also kangaroos, which have dental drift. However, because of the compressive force of erupting teeth, the perfect anchoring of teeth is a necessary prerequisite to avoid dental disorders. Some traversodontids, tritylodontids, manatees, and the pygmy rock-wallaby have elongated roots, which prevent such problems. The silvery mole-rat is the only one chisel-tooth digger among bathyergids to have very high-crowned teeth, which are also cone-shaped as in some traversodontids (e.g., *Boreogomphodon*^†^, Liu & Sues, 2010). That characteristics has probably facilitated the mechanical superimposition of CDR, whatever its function, as well as mesial drift also observed in other high-crowned mole-rats (e.g., *Bathyergus*).

The biological origins of CDR in mammals remains however to discover. Even if mammals having CDR show striking morphological convergences with non-mammalian synapsid groups, it seems unlikely that the same developmental pathways permitting new generation of teeth distally were reactivated after such a very long period of time (Gomes Rodrigues et al., 2011). Nonetheless, specific biological parameters probably shared between non-mammalian-synapsids and these mammals might have favoured the acquisition of CDR. For instance, it should be noticed that these mammals have low basal metabolic rates (Gallivan & Best, 1980; Janis & Fortelius, 1988; Mc Nab, 2005; Zelová et al., 2007), as show “reptiles” (i.e., Sauropsida), which generally have continuous generation of teeth, even if not horizontal. Direct or indirect relations between CDR and such biological parameters cannot be demonstrated for the moment. In any event, these troubling “reptile-like” similarities clearly need deeper considerations, and the silvery mole-rat could appear as an appropriate candidate for further researches. This will contribute to a better understanding of the origins of the mammalian CDR, but also to the gathering of new data on the biological mechanisms leading to the reduction of the dentition at the origin of mammals.
